# Effectiveness of Occupational Therapy-Based Intervention on Gross Motor Function and Independence in Activities of Daily Living in Children with Cerebral Palsy: A Systematic Review with Meta-Analysis

**DOI:** 10.3390/jcm14217624

**Published:** 2025-10-27

**Authors:** Diego Fernandez-Cardenas, Celia Sánchez-Gomez, Edgar Vásquez-Carrasco, Jordan Hernandez-Martinez, Joaquín Pérez-Cárcamo, Cristian Sandoval, Pablo Valdés-Badilla, Eduardo Carmine-Peña, Constanza Lorca, Eduardo Fernández-Rodríguez

**Affiliations:** 1 School of Occupational Therapy, Health Sciences Faculty, Catholic University of Maule, Talca 3530000, Chile; dfernandezc@ucm.cl; 2 Department of Developmental and Educational Psychology, University of Salamanca, 37008 Salamanca, Spain; 3 Institute of Biomedical Research of Salamanca (IBSAL), 37007 Salamanca, Spain; edujfr@usal.es; 4 School of Occupational Therapy, Faculty of Psychology, Universidad de Talca, Talca 3465548, Chile; 5 Centro de Investigación en Ciencias Cognitivas, Faculty of Psychology, Universidad de Talca, Talca 3465548, Chile; 6 VITALIS Longevity Center, Universidad de Talca, Talca 3465548, Chile; 7 Department of Physical Activity Sciences, Universidad de Los Lagos, Osorno 5290000, Chile; jordan.hernandez@ulagos.cl; 8 G-IDyAF Research Group, Department of Physical Activity Sciences, Universidad de Los Lagos, Osorno 5290000, Chile; joaquinalejandro.perez@alumnos.ulagos.cl; 9 Escuela de Tecnología Médica, Facultad de Salud, Universidad Santo Tomás, Los Carreras 753, Osorno 5310431, Chile; cristian.sandoval@ufrontera.cl; 10 Departamento de Medicina Interna, Facultad de Medicina, Universidad de La Frontera, Temuco 4811230, Chile; 11 Department of Physical Activity Sciences, Faculty of Education Sciences, Universidad Católica del Maule, Talca 3530000, Chile; valdesbadilla@gmail.com; 12 Sports Coach Career, Faculty of Life Sciences, Universidad de Viña del Mar, Viña del Mar 2520000, Chile; 13 Carrera de Medicina, Facultad de Medicina, Universidad de La Frontera, Temuco 4811230, Chile; e.carmine01@ufromail.cl; 14 Departamento de Ciencias Básicas, Facultad de Medicina, Universidad de La Frontera, Temuco 4811230, Chile; 15 Department of Nursing and Physiotherapy, University of Salamanca, 37007 Salamanca, Spain

**Keywords:** child, neurology, rehabilitation, technology

## Abstract

**Background/Objectives:** Children with cerebral palsy (CP) commonly present impairments in gross motor function and limitations in activities of daily living (ADLs), which negatively impact independence and quality of life. Identifying effective rehabilitation strategies is essential to promote functional development. To evaluate the effectiveness of occupational therapy (OT) interventions on gross motor function and independence in ADLs among children with CP. **Methods:** Seven electronic databases were searched through August 2025. The review protocol was registered in PROSPERO (CRD42025634706) and conducted in accordance with PRISMA guidelines. Methodological quality and certainty of evidence were assessed using the Oxford Centre for Evidence-Based Medicine scale, the Risk of Bias 2 (RoB 2) tool, and GRADEpro. Randomized controlled trials reporting OT interventions targeting gross motor and ADL outcomes were included. **Results:** Of 594 identified records, 14 studies met the inclusion criteria. Meta-analysis indicated that OT interventions significantly improved gross motor function (GMFM-66; ES = 0.32 [0.01–0.63], *p* = 0.04), mobility (PEDI-Mobility; ES = 0.46 [0.05–0.87], *p* = 0.02), and occupational performance (COPM-Performance; ES = 2.63 [1.14–4.11], *p* = 0.001) and satisfaction (COPM-Satisfaction; ES = 2.17 [0.82–3.51], *p* = 0.002). No significant changes were observed in self-care (PEDI-Self-Care; ES = 0.19 [−0.14–0.53], *p* = 0.26). **Conclusions:** Evidence suggests that OT interventions effectively enhance gross motor function, mobility, and occupational performance in children with CP. These results support the integration of OT within pediatric rehabilitation programs to optimize functional outcomes.

## 1. Introduction

Cerebral palsy (CP) represents the leading cause of physical disability in childhood, with a global prevalence estimated at roughly 2–3 cases per 1000 live births [1]. It is characterized by impairments in movement and postural control, often accompanied by disturbances in balance [2]. The earlier belief that cerebral asphyxia was the predominant cause of CP has been replaced by evidence supporting a complex, multifactorial origin that includes prenatal, perinatal, and postnatal risk factors [3].

Advancements in the classification of CP now allow for categorization based on motor type, functional severity, topographical distribution, and underlying etiology [4,5]. Clinical manifestations evolve over time but are typically evident between the ages of 3 and 5 years [5]. Management requires a multidisciplinary approach, integrating neurological rehabilitation with the treatment of comorbidities such as epilepsy, and sensory, cognitive, and gastrointestinal disorders, necessitating the involvement of a wide range of healthcare professionals [6].

Given that CP is categorized by impairments in gross motor performance, postural control, hand dexterity, communication, and progressive musculoskeletal limitations, these deficits lead to functional dependence and restrictions in activities of daily living (ADLs) [6]. Intervention outcomes are frequently evaluated through standardized classification frameworks such as the Manual Ability Classification System (MACS), the Gross Motor Function Classification System (GMFCS), and the Communication Function Classification System (CFCS) [7]. These tools demonstrate strong psychometric properties. The GMFCS demonstrates high inter- and intra-rater reliability [8], while the MACS reports intra-rater reliability of 0.97 and inter-rater reliability of 0.94 (95% CI: 0.89–0.97) [9,10]. Evidence of construct validity has been demonstrated through strong inter-scale correlations, specifically between GMFCS and MACS, and between MACS and CFCS; indicating a consistent relationship among motor, manual, and communication domains [11,12].

Measurement stability is confirmed by low standard error of measurement (SEM) values for the GMFCS and MACS, although the small effect sizes suggest limited sensitivity to detect clinically significant changes post-intervention [11,12]. Moreover, results from the Egger’s test suggested the presence of publication bias in studies involving the MACS (*p* = 0.041) [13]. Gross motor performance is often quantified with the Gross Motor Function Measure-88 (GMFM-88) [14], and self-care or daily activity independence is typically examined through the WeeFIM scale [15].

Early diagnosis of CP is fundamental to assessing therapeutic effectiveness and fostering favorable developmental outcomes [16]. Evidence-based motor interventions, such as constraint-induced movement therapy (CIMT), have resulted in measurable improvements in unimanual and bimanual motor control [17]. Additionally, enriched environments have been shown to facilitate motor and cognitive development [18]. Targeted therapies like hippotherapy enhance gross motor skills and functional independence [19], while neurodevelopmental therapy supports both neural and motor maturation [20]. Moreover, virtual reality-based interventions have shown promising results compared with conventional rehabilitation methods in improving motor outcomes [21]. However, few reviews have examined these variables comprehensively [22].

Occupational therapy (OT) contributes significantly to the management of CP by encouraging participation in meaningful daily activities that promote overall health and well-being [23]. In children with CP, OT focuses on enhancing skills necessary for ADLs, promoting independence, and improving quality of life [24]. OT is defined as a health profession aimed at enabling participation in activities that support health and well-being. It addresses physical, emotional, social, and environmental factors that influence occupational performance [25]. In pediatric populations, evidence-based OT interventions include bimanual training, CIMT, goal-directed programs, early intervention, assistive technology, and virtual rehabilitation [16]. These strategies prioritize functional outcomes and emphasize collaboration with caregivers to enhance the child’s autonomy and engagement in meaningful activities [26].

Therefore, the objective of this systematic review and meta-analysis was to evaluate the effectiveness of occupational therapy-based intervention on gross motor function and ADL independence in children with cerebral palsy.

## 2. Materials and Methods

### 2.1. Protocol and Registration

The present systematic review with meta-analysis adhered to Cochrane methodological standards [27] and complied with PRISMA reporting requirements [28] (Table S1). The protocol was registered in PROSPERO (CRD42025634706) on 17 January 2025.

### 2.2. Eligibility Criteria

Eligible studies comprised randomized controlled trials (RCTs) published from January 2024 through August 2025, without restrictions regarding language or publication status. Non-randomized designs, descriptive reports, reviews, and non-peer-reviewed sources were excluded. The inclusion process was structured using the PICOS framework (Population, Intervention, Comparator, Outcome, Study Design), as presented in Table 1.

### 2.3. Information Search Process and Database

Data sources included seven databases: PubMed (National Center for Biotechnology Information), Web of Science Core Collection, Scopus, EBSCOhost, ProQuest, Cochrane Library, and CINAHL. The search terms included: (“Cerebral Palsy” OR “Ataxic” OR “Cerebral Palsy Spastic” OR “Diplegic Cerebral Palsy” OR “Spastic Quadriplegic” OR “dystonic cerebral palsy”) AND (“Motor skills” OR “Gross Motor Skills” OR “Motor Coordination” OR “Large Muscle Movement” OR “Gross Motor Abilities” OR “Motor Control” OR “Body Movement Coordination” OR “Postural Control” OR “Physical Coordination” OR “Movement Efficiency” OR “Motor Proficiency” OR “Locomotor Skills”) AND (“Occupational Therapy” OR “Occupational Therapy Interventions” OR “Occupational Therapist”). Validation of the study selection and eligibility criteria was performed by an independent expert to confirm the relevance of the included publications. The independent expert met two criteria: holding a PhD in health sciences and having published peer-reviewed research in Journal Citation Reports^®^-indexed journals on occupational therapy-based intervention addressing motor function and ADL performance. To minimize bias, the approach used in the search was not revealed to him. A follow-up search was conducted within the databases to detect any retractions or corrections related to the studies included in the review.

### 2.4. Study Selection Process and Data Collection

Studies were organized and managed using Mendeley Reference Manager (version 2.116.1). The search process was independently undertaken by two authors (D.F.-C. and E.V.-C.) to minimize selection bias, assessing titles, abstracts, and full texts and removing duplicates. No discrepancies were detected during this review. Articles deemed eligible were further examined in detail, and reasons for excluding records that did not satisfy the inclusion criteria were documented and reported in the PRISMA flow diagram. A third reviewer (P.V.-B.) independently audited the entire selection and data extraction process.

### 2.5. Methodological Quality Assessment

The quality appraisal and evidence grading of the included studies followed the standards established by the Oxford Centre for Evidence-Based Medicine scale [29]. Inclusion was restricted to level 1a studies, corresponding to RCTs, with all lower evidence levels (1b–5) excluded from consideration. In addition, when methodological limitations such as bias risk, inconsistency, imprecision, lack of clarity, or suspected publication bias were identified, RCTs were reappraised and assigned a lower evidence level accordingly [29].

### 2.6. Data Collection Process

For each study included in the review, relevant information was extracted using a customized data collection form developed in alignment with Cochrane Collaboration standards [30]. This process was carried out using Microsoft Excel^®^ (version 16.81). Two researchers (D.F.-C. and E.V.-C.) independently performed the extraction and compared their results to ensure accuracy. A third reviewer (P.V.-B.) supervised the entire procedure. Data extraction included bibliographic (title, authors, year, and country) and methodological details (design, evidence level, and bias assessment), participant and sampling characteristics, eligibility criteria, study context, intervention and control descriptions, and outcomes related to gross motor performance, ADL independence, and overall study conclusions.

### 2.7. Risk of Bias

To assess the risk of bias in the selected studies, the Cochrane tool for RCTs (RoB 2.0) was used [27]. This approach covered five key aspects: randomization, deviations in the intervention, incomplete outcome data, outcome assessment and selecting reported outcomes. The analysis was carried out collaboratively by 2 authors (D.F.-C. and E.V.-C.) and subsequently verified by an independent reviewer (P.V.-B.).

### 2.8. Meta-Analysis Measures

The study employed a meta-analysis approach, with the methodology registered in PROSPERO (CRD42025634706). Effect sizes were expressed as standardized mean differences (SMDs), computed for each randomized controlled trial comparison to determine mean group differences using Review Manager software (RevMan 5.4; The Cochrane Collaboration, London, UK). To account for small-sample bias, effect sizes were expressed as Hedges’ g, a corrected form of the standardized mean difference. A significance level of *p* < 0.05 was adopted for all analyses [31]. A random-effects model, using the Der Simonian-Laird method, was applied to estimate and combine the SMD and mean differences across outcomes, such as gross motor function and ADLs, comparing experimental and control groups before and after the intervention [32]. This model assumes variation in true intervention effects across studies due to factors like intervention type or duration, reflecting diverse effect sizes among populations. Results were pooled when at least three studies provided consistent findings [33]. Heterogeneity across studies was evaluated using the Cochrane Q test [34] and the I^2^ statistic, with values below 25%, between 25 and 49%, and above 50% representing low, moderate, and high heterogeneity, respectively [32]. In addition, Egger’s regression test was applied to identify potential small-study effects and publication bias [35].

### 2.9. Certainty of Evidence

The GRADE (Grading of Recommendations, Assessment, Development, and Evaluation) system was applied to assess the certainty and methodological robustness of the evidence across the included studies [27,36], classifying the levels of evidence as high, moderate, low, or very low. Since all studies were RCTs, a high level of certainty was used as the starting point, which was subsequently adjusted if problems such as risk of bias, lack of consistency, imprecision, lack of accuracy, opacity in results, or publication bias were identified. Two authors (D.F.-C. and E.V.-C.) carried out this assessment independently, and any differences in the results were resolved by consensus with the participation of a third author (P.V.-B.).

## 3. Results

### 3.1. Study Selection

**Figure 1:**
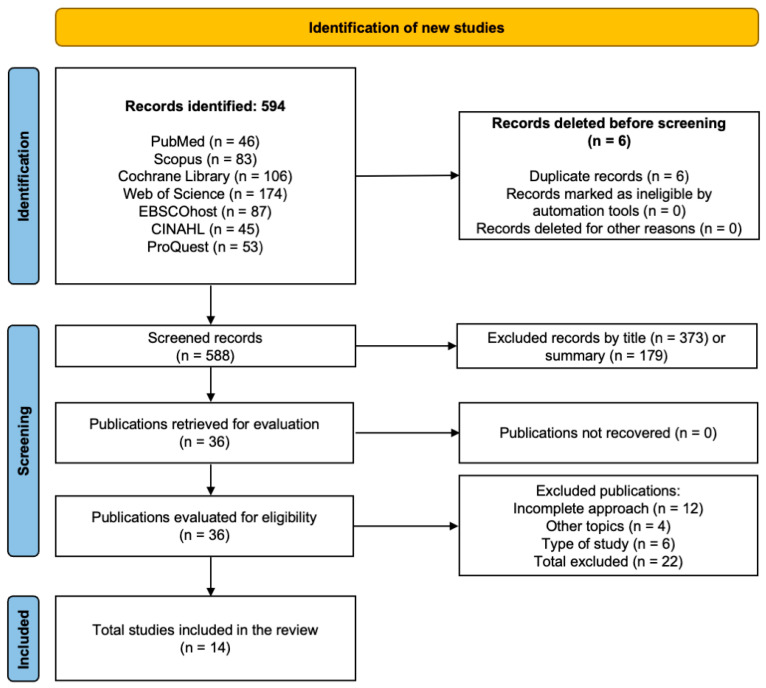
PRISMA flow diagram illustrating the study selection process for the systematic review.

The database search yielded 594 studies in total, identified from PubMed, ProQuest, Scopus, Web of Science, Cochrane Library, and EBSCOhost CINAHL. Following the exclusion of six duplicates, 588 studies remained for the screening process. Of these, 552 were excluded based on title and abstract review for not meeting the eligibility criteria, leaving 36 articles for full-text assessment. Following a thorough evaluation, 22 articles were excluded for the following reasons: 12 lacked a complete focus on the topic, 4 addressed unrelated subjects, and 6 did not meet the required study design criteria. Ultimately, 14 studies were retained for inclusion in the review [16,37,38,39,40,41,42,43,44,45,46,47,48,49], providing evidence on the efficacy of interventions to improve gross motor abilities and support independence in ADLs for children with CP. The selection pathway is presented in Figure 1.

### 3.2. Methodological Quality

This systematic review and meta-analysis comprised studies of high methodological quality, all of which were RCTs [16,37,38,39,40,41,42,43,44,45,46,47,48,49], corresponding to level 1a evidence according to the Oxford classification.

### 3.3. Risk of Bias

**Figure 2:**
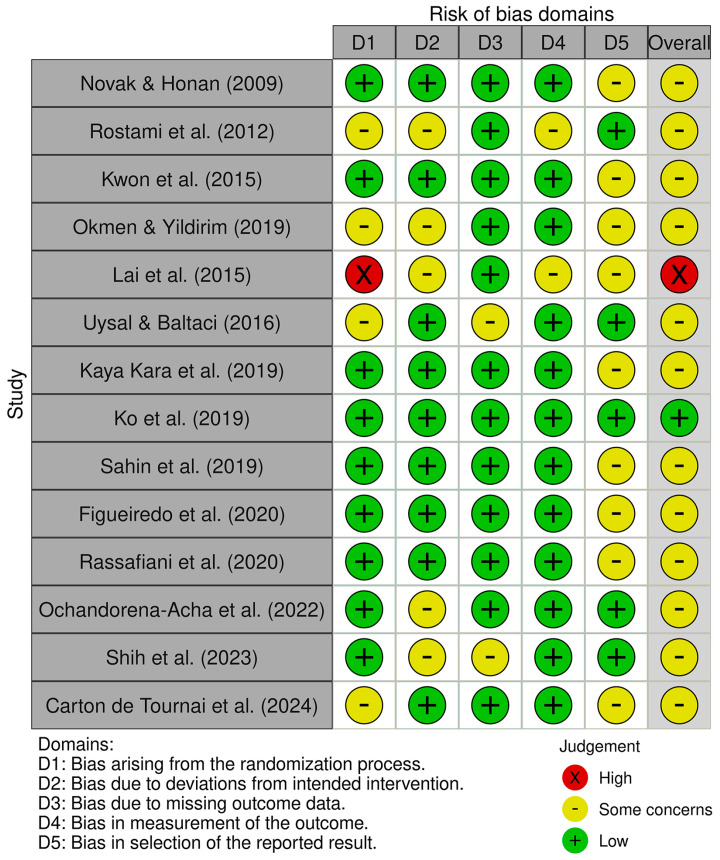
Risk of bias tool: traffic light chart [26,37,38,39,40,41,42,43,44,45,46,47,48,49].

**Figure 3:**
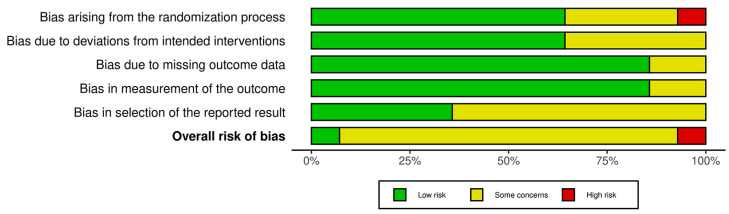
Risk of bias tool: summary table by domain.

The risk of bias assessment identified one study with low risk [42], twelve with some concerns [16,37,38,39,41,43,44,45,46,47,48,49], and one with high risk [40]. These results collectively suggest a moderate concern for bias across the reviewed evidence. Figure 2 and Figure 3 present a visual overview of these findings.

### 3.4. Characteristics of the Studies

Of the 14 studies reviewed, various interventions demonstrated positive effects on gross motor function and ADLs in children with CP. Virtual reality interventions, like Kinect, were particularly effective for motor function and independence [39,48]. Game-based interventions, such as the Nintendo Wii™, improved balance and occupational activities [41]. Combined approaches, like aquatic therapy with functional exercises and intensive programs like Hand-Arm Bimanual Intensive Therapy, also yielded positive results [40,45]. Additionally, therapies such as hippotherapy, mirror therapy with muscle strength exercises, and early interventions like HABIT-ILE significantly enhanced motor skills, balance, and bimanual performance [38,43,49]. These findings highlight the value of diverse, individualized interventions in improving both functional abilities and overall well-being in children with CP. The summary of the characteristics of each study and their main results is described in Table 2.

### 3.5. Sample Characteristics

A total of 510 pediatric participants with CP were included in the studies analyzed within this systematic review and meta-analysis. The mean age was 7.6 years, with a range varying between 1.1 years (13 months) and 12 years. Sample sizes varied from a minimum of 14 participants [48] to a maximum of 91 participants [38], considering the diverse interventions implemented and the outcomes reported in each case.

### 3.6. Administered Dosages and Executed Interventions

The reviewed programs included activities aimed at improving gross motor function and independence in ADLs, varying in duration, frequency, and intensity. Several studies implemented 8-week interventions with 2 weekly sessions, such as Shih et al. [48] with Kinect-based and therapist-based CIMT (135 min/session), Ko et al. [42] with task-oriented training (30 min/session), and Sahin et al. [44] with virtual reality therapy via Kinect (45 min/session), all at moderate intensity. More intensive interventions included Figueiredo et al. [45] with HABIT (three weeks, five sessions of 360 min/week) and Carton de Tournai et al. [49] with HABIT-ILE (2 weeks, 5 sessions of 300 min/week). Different strategies were used, such as mirror therapy and strength exercises [43], pediatric aquatic therapy [40], treadmill training combined with virtual reality [47], and hippotherapy [38], with intensities ranging from moderate to high.

### 3.7. Gross Motor Function

**Figure 4:**
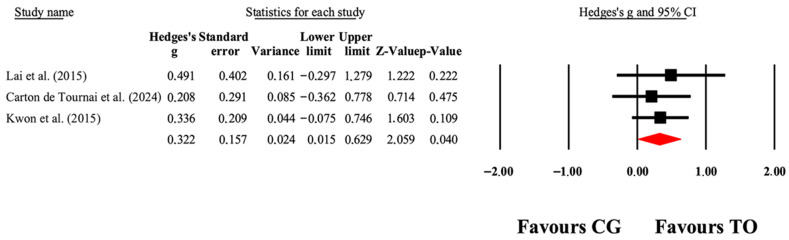
The forest plot illustrates the changes in GMFM-66 scores among patients with cerebral palsy who participated in occupational therapy compared to those assigned as controls. The values presented represent effect sizes (Hedges’ g) along with 95% CI. The size of the squares in the plot indicates the statistical weight of each study [38,40,49].

Overall, the meta-analytic results favored OT interventions for improving gross motor function (Table 3). As illustrated in Figure 4, small to large effect sizes (ES = 0.01–0.63) indicated significant gains in GMFM-66 outcomes. Funnel plot of standard error by Hedge’s g can be found in Figure S1.

### 3.8. Activities of Daily Living

**Figure 5:**
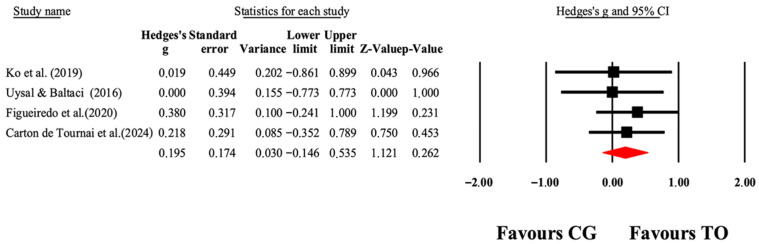
The forest plot illustrates the changes in PEDI-Self-Care scores among patients with cerebral palsy participating in occupational therapy compared to those assigned as controls. The values presented represent effect sizes (Hedges’ g) along with 95% CI. The size of the squares in the plot indicates the statistical weight of each study [41,42,45,49].

**Figure 6:**
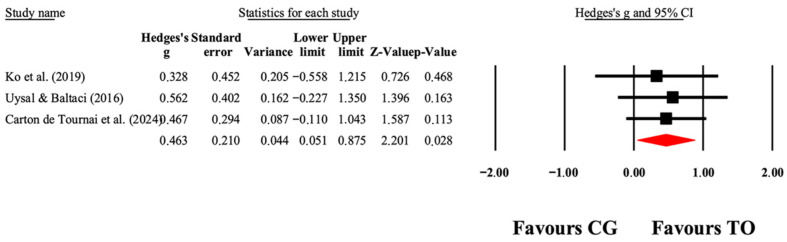
The forest plot illustrates the changes in PEDI-Mobility in patients with cerebral palsy participating in occupational therapy compared with patients with cerebral palsy assigned as controls. Values shown are effect sizes (Hedges’ g) with 95% CI. The size of the squares plotted reflects the statistical weight of each study [41,42,49].

**Figure 7:**
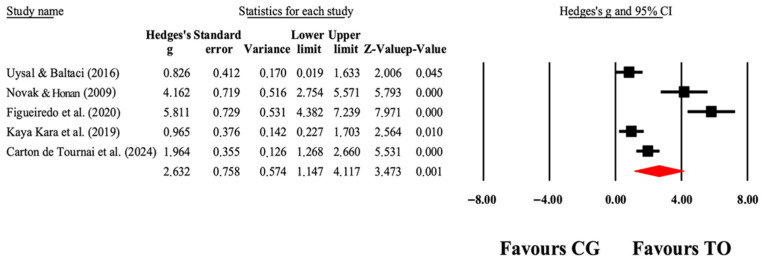
The forest plot illustrates the changes in COPM-Performance in patients with cerebral palsy participating in occupational therapy compared with patients with cerebral palsy assigned as controls. Values shown are effect sizes (Hedges’ g) with 95% CI. The size of the squares plotted reflects the statistical weight of each study [26,41,43,45,49].

**Figure 8:**
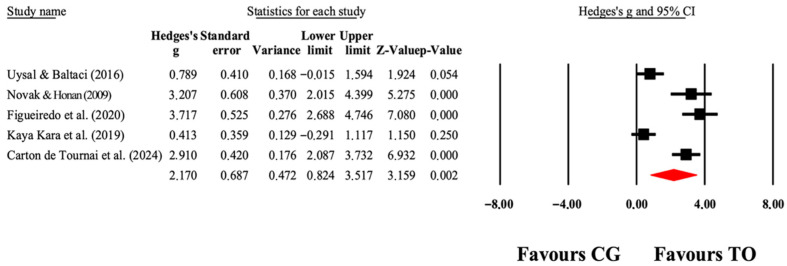
The forest plot illustrates the changes in COPM-Satisfaction in patients with cerebral palsy participating in occupational therapy compared with patients with cerebral palsy assigned as controls. Values shown are effect sizes (Hedges’ g) with 95% CI. The size of the squares plotted reflects the statistical weight of each study [26,41,43,45,49].

The overall effects of OT interventions on the independence of ADLs (as assessed by functional capacity, occupational performance, and satisfaction) are shown in Table 4. Forest plots are shown in Figure 5, Figure 6, Figure 7 and Figure 8. Small to large effect sizes (ES = 0.19 to 2.63) showed significant differences related to the OT intervention evidenced in PEDI-Mobility, COPM-Performance and COPM-Satisfaction, with no significant differences found in PEDI-Self Care. Funnel plot of standard error by Hedge’s g for Figure 5, Figure 6, Figure 7 and Figure 8 can be found in Figures S2–S5, respectively.

### 3.9. Certainty of Evidence

Evidence concerning OT interventions in children with CP provides moderate certainty for benefits in gross motor function and ADLs. However, certain studies revealed methodological weaknesses that suggest some degree of bias, they do not raise any issues regarding inconsistency or precision, which supports their clinical relevance (Table 5).

### 3.10. Effects Adverse and Adherence

The pooled data from the included studies revealed a 76.4% adherence rate and no reported adverse effects. These results indicate that the interventions were both feasible and well tolerated in children with CP, reinforcing their suitability for broader clinical application.

## 4. Discussion

### 4.1. Gross Motor Function

The meta-analysis identified significant gains in gross motor function assessed via the GMFM-66 (*p* = 0.04). Supporting these findings, Szturm et al. [50] reported that a 12-week game-based intervention significantly improved balance (*p* = 0.03) and GMFM-88 subdomains of standing (*p* = 0.05) and walking/running/jumping (*p* = 0.03). Likewise, Rastgar Koutenaei et al. [51] observed greater post-intervention and 9-week GMFM-88 improvements following Swiss ball training compared with stable surface exercises (*p* < 0.001). Matusiak-Wieczorek et al. [52] observed that hippotherapy enhanced trunk control, with significant gains in the group receiving two sessions per week (*p* = 0.005), whereas improvements with one session weekly were not significant (*p* = 0.028). Conversely, Jha et al. [53] found that virtual reality combined with physical therapy improved GMFM-88 scores within groups but not between groups (*p* = 0.519). Overall, these findings highlight the importance of individualized interventions to optimize motor function in children with CP.

### 4.2. Activities of Daily Living

Significant improvements were identified in ADLs following occupational therapy-based intervention including occupational performance and satisfaction (COPM-Performance, *p* = 0.001; COPM-Satisfaction, *p* = 0.002) and mobility (PEDI-Mobility, *p* = 0.02). However, no significant change was observed in self-care outcomes (PEDI Self-Care, *p* = 0.26). Consistent with the meta-analytic results, Choi et al. [54] observed significant ADL gains following four weeks of virtual reality rehabilitation compared with conventional occupational therapy (PEDI-CAT, *p* < 0.01), with greater improvements among children with severe motor limitations. Consistently, Yang et al. [55] found that a rehabilitation program grounded in the ICF framework significantly improved ADLs after three months (*p* < 0.001), showing superior outcomes compared with traditional therapy. Özen et al. [56] observed significant ADL improvements following functional electrical stimulation in children with spastic diplegic CP (*p* = 0.004). Conversely, Jha et al. [53] reported that both virtual reality and control groups improved on the WeeFIM (*p* < 0.001 and *p* < 0.05, respectively), but without between-group differences (*p* = 0.33). Overall, these findings suggest that OT-based rehabilitation can effectively enhance ADLs in children with CP, though outcomes vary depending on intervention type and motor severity.

### 4.3. Limitations and Strengths

This systematic review and meta-analysis acknowledge several limitations: (i) the restricted number of included studies may have reduced the analytical breadth, (ii) although methodological rigor was generally high, twelve studies raised some concerns regarding potential bias; and (iii) small sample sizes and the heterogeneity of intervention protocols limit the ability to draw definitive conclusions.

Nevertheless, this review also has notable strengths. It exclusively included RCTs. Furthermore, it incorporated innovative interventions, such as virtual reality and functional electrical stimulation, which reflect emerging therapeutic trends. The included studies reported significant improvements in motor function and ADLs. Additionally, the combined sample size of 510 participants contributes to the external validity of the findings.

### 4.4. Practical Applications

This systematic review and meta-analysis highlight the efficacy of occupational therapy-based intervention in improving gross motor function and ADLs among children with CP. Evidence supports the effectiveness of modalities such as virtual reality, game-based programs, intensive bimanual hand and arm therapy, hippotherapy, mirror therapy, and aquatic therapy [39,41,44,45,47,48]. Collectively, these findings advocate for individualized, evidence-based rehabilitation strategies that consider each child’s functional profile and promote both motor improvement and overall well-being. From a translational standpoint, these results emphasize the need to integrate multimodal, client-centered interventions into clinical practice. Future research should prioritize high-quality comparative trials to determine the relative effectiveness of these approaches, explore long-term functional outcomes, and evaluate their feasibility and scalability across diverse rehabilitation contexts.

## 5. Conclusions

OT interventions in children with CP showed significant improvements in gross motor function, measured with GMFM-66, and in independence in ADL, assessed with COPM-Performance, COPM-Satisfaction, and PEDI-Mobility. However, no significant differences were observed in the results measured with PEDI-Self Care.

## Figures and Tables

**Table 1:** Selection criteria used in the systematic review.

Category	Inclusion	Exclusion
Population	Research focused on children diagnosed with cerebral palsy, aged between 1 and 12 years.	Studies whose focus is a pathology other than cerebral palsy or that involve a population outside the childhood age range.
Intervention	Studies that address occupational therapy-based intervention for four weeks or more.	Studies that do not have occupational therapy-based intervention as their primary focus.
Comparison	Trials incorporating active or inactive control conditions were considered.	Studies lacking a control arm or involving untreated controls were not included.
Outcomes	Studies that present results in gross motor functions and ADLs, assessed using validated tools.	Incomplete datasets, defined by missing baseline values or follow-up outcomes.
Study design	Experimental design studies (randomized controlled clinical trials).	Non-randomized or observational designs (cross-sectional, retrospective, or prospective controlled studies).

**Table 2:** Summary table of results characteristics.

Study	Country	Study Design	Sample	Groups (n)	Average Age (Years)	Types of Intervention and Control Group	Volume Training			Training Intensity Assessment	Assessment	**Main Results**
							Weeks	Frequency (Sessions/ Week)	Session Duration (min)			
[26]	AU	RCT	Children with cerebral palsy	8-week OTHP intervention group: 12 4-week OTHP intervention group: 12 CG: 12	7.7 years old	OTHP (8-week or 4-week) intervention: Parent-led home occupational therapy program with support from therapists CG: Group without OTHP intervention	8	17.5	16.5	Moderate	COPM-P; COPM-S; GAS and QUEST	COPM-P: *p* = 0.01 COPM-S: *p* = 0.01 GAS: *p* = 0.01 QUEST: *p* = 0.02
[37]	IR	RCT	Children with spastic hemiplegic cerebral palsy	Home treatment group: 7 Clinic treatment group: 7	74 months	Home treatment group: Modified CIMT at home Clinic treatment group: Modified CIMT in a clinical setting	10	3	90	High, with intensive and prolonged training sessions for the affected hand	PMAL and BOT-2	PMAL: AOU subscale: *p* = 0.001 QOM subscale: *p* = 0.001 BOT-2: Subtest 8 (Upper Limb Speed and Dexterity): *p* = 0.001
[38]	KR	RCT	Children with CP	EG: 45 CG: 46	5.8 ± 1.8 years	EG: Hippotherapy (30 min, twice weekly) + conventional therapy CG: Home-based aerobic exercises + conventional therapy	8	2	30	Moderate to high	GMFM-66; GMFM-88 and PBS	GMFM-66: *p* < 0.01 GMFM-88: Total: *p* < 0.01 Dimension B (Sitting): *p* < 0.01 Dimension C (Crawling and Kneeling): *p* < 0.01 Dimension D (Standing): *p* < 0.01 Dimension E (Walking, Running, and Jumping): *p* < 0.01 PBS: *p* < 0.01
[39]	TR	RCT	Children with cerebral palsy	EG: 21 Control: 20	8.49 years	EG: VR therapy + conventional treatment Control: Conventional treatment only	4	3	60	Moderate	BFMF, GMFCS and FMS	BFMF: *p* < 0.001 GMFCS: *p* = 0.005 FMS: *p* = 0.002
[40]	TW	Prospective, blind, quasi-experimental study.	Children with spastic cerebral palsy	EG: 11 CG: 13	7.1 ± 2.8 years	EG: Pediatric aquatic therapy (in a pool with temperatures of 33–36 °C, 2 times a week) CG: Conventional therapy	12	2	60	Moderate	GMFM-66 and PACES	GMFM-66: *p* = 0.007 PACES: *p* = 0.015
[41]	TR	RCT	Children with spastic hemiplegic cerebral palsy (GMFCS level I and II).	EG: 12 CG: 12	EG: 9.13 years CG: 10.11 years	EG: Training with Nintendo Wii™ + traditional therapy CG: Traditional therapy only	12	2	30	Moderate	COPM; PEDI and PBS	COPM: Performance: *p* = 0.007 PEDI: Self-care: *p* = 0.004 Mobility: *p* = 0.005 Total: *p* = 0.003 PBS: *p* = 0.006
[42]	KR	RCT	Children with spastic cerebral palsy	TOT-group: 9 Comparison group: 9	TOT-group: 4.9 ± 1.1 years Comparison group: 5.1 ± 1.5 years	TOT-group: Group task-oriented training Comparison group: Traditional physical and OT	8	2	30	Moderate	GMFM-88; BOT-2 and PEDI	GMFM-88: Standing subscale (*p* = 0.03) Walking/Running/Jumping subscale (*p* = 0.02) BOT-2: Manual Dexterity subscale (*p* = 0.04) PEDI: Social Function subscale (*p* = 0.04)
[43]	TR	RCT	Children with USCP	EG: 17 CG: 17	12 years	EG: Mirror therapy combined with strength and power exercises CG: Occupational therapy	12	3	60	Moderate to high	QUEST; COPM and Isometric strength with handheld dynamometer	QUEST: Dissociated Movements: *p* < 0.001 Grasp: *p* < 0.001 Weight Bearing: *p* = 0.006 Protective Extension: *p* = 0.061 Total: *p* = 0.001 COPM: Performance: *p* < 0.001 Satisfaction: *p* < 0.001 Total: *p* < 0.001 Muscle Strength (Handheld Dynamometer): Affected Upper Extremity: Elbow Flexors: *p* < 0.001 Elbow Extensors: *p* = 0.002 Unaffected Upper Extremity: Elbow Flexors: *p* < 0.001 Elbow Extensors: *p* < 0.001
[44]	TR	RCT	Children with USCP	EG: 30 CG: 30	EG: 10.5 ± 3.62 years CG: 10.06 ± 3.24 years	EG: VR therapy through Kinect + TOT CG: TOT	8	2	45	Moderate	BOTMP-SF and WeeFIM	BOTMP-SF: *p* < 0.001 WeeFIM: *p* < 0.001
[45]	BR	RCT	Children with BCP	EG: 19 CG: 20	9.6 ± 3.9 years	EG: HABIT CG: Customary Care	3	45	360	Intensive	COPM; PEDI; BoHA; JTTHF and BBT	COPM, PEDI: Improved daily functioning in HABIT group BoHA: No significant improvement in bimanual performance JTTHF: No significant improvement in non-dominant hand dexterity BBT: Improved dexterity in dominant hand (*p* < 0.05)
[46]	IR	RCT	Children with bilateral spastic cerebral palsy (level I and II in the GMFCS classification)	EG: 12 CG: 12	EG: 8.41 ± 1.00 years CG: 9.18 ± 1.66 years	EG: Occupational therapy plus active vestibular interventions CG: Regular occupational therapy without active vestibular interventions	6	3	45	Moderate	PBS; BOT-2 and ASK	PBS: The results were not significant in the control group (*p* = 0.256) BOT-2 (Balance Subtest): No significant differences were found in the control group (*p* > 0.05) ASK: No significant improvements were observed in the control group (*p* = 0.59)
[47]	SP	RCT	Children with spastic cerebral palsy	EG: 15 CG: 15	The participants are between 4 and 12 years old	EG: Treadmill training + VR therapy CG: Treadmill training alone	2	5	30	High, with gradual increase in treadmill speed in each session	6MWT; BBS; GMFM-66; WeeFIM; PedsQoL and CPQ	6MWT: *p* < 0.00 BBS: *p* = 0.004 GMFM-66: *p* = 0.005 WeeFIM: *p* = 0.03 PedsQoL: *p* = 0.02 CPQ: *p* = 0.01
[48]	TW	RCT	Children with UCP	Kinect-based CIMT: 14 Therapist-based CIMT: 15	8 ± 2.2 years old	Kinect-based CIMT and Therapist-based CIMT (both constraint-induced movement therapy interventions).	8	2	145	Moderate	UE; PMAL-R; nED; nTD and TCS	Upper Extremity Motor Control (UE): *p* > 0.05 Daily Motor Function (PMAL-R): *p* > 0.05 Trunk Motor Control (nTD, nED, TCS): nED before PV (*p* = 0.036) nTD after PV (*p* = 0.021) TCS before PV (*p* = 0.033) TCS after PV (*p* = 0.019)
[49]	BE	RCT	Infants with UCP	EG: 24 CG: 22	~13.3 ± 4.1 months	EG: Baby Hand-Arm Bimanual Intensive Therapy Including Lower Extremities (HABIT-ILE) CG: Usual motor activities	2	5	300	High	Mini-AHA; COPM; GMFM-66 and PEDI-CAT	Mini-AHA: *p* = 0.008 COPM: Performance: *p* < 0.001 Satisfaction: *p* < 0.001 GMFM-66: *p* < 0.001 PEDI-CAT: Daily Activity: *p* < 0.001 Mobility: *p* < 0.001

6MWT: 6-Minute Walk Test; ADAS-K-cog: Korean version of the Alzheimer’s Disease Assessment Scale-Cognitive Subscale; ADL: Activities of Daily Living; AOU: Amount of Use; AU: Australia; BBT: Box and Block Test; BCP: Bilateral Cerebral Palsy; BE: Belgium; BFMF: Bimanual Fine Motor Function; BoHA: Both Hands Assessment; BBS: Berg Balance Scale; BOT-2: Bruininks-Oseretsky Test of Motor Proficiency; BOTMP-SF: Bruininks-Oseretsky Test of Motor Proficiency-Short Form; CG: Control Group; CIMT: Constraint-Induced Movement Therapy; COPM: Canadian Occupational Performance Measure; COPM-P: Canadian Occupational Performance Measure-Performance; COPM-S: Canadian Occupational Performance Measure-Satisfaction; CP: Cerebral Palsy; CPQ: Cerebral Palsy Quality of Life Questionnaire; EG: Experimental Group; FMS: Functional Mobility Scale; GAS: Goal Attainment Scaling; GMFCS: Gross Motor Function Classification System; GMFM-66: Gross Motor Function Measure-66; GMFM-88: Gross Motor Function Measure-88; HABIT: Hand-Arm Bimanual Intensive Therapy; HABIT-ILE: Hand-Arm Bimanual Intensive Therapy Including Lower Extremities; IR: Iran; JTTHF: Jebsen-Taylor Test of Hand Function; KR: Korea; Mini-AHA: Mini-Assisting Hand Assessment; nED: normalized Endpoint Deviation; nTD: normalized Trunk Displacement; OT: Occupational Therapy; OTHP: Occupational Therapy Home Program; PACES: Physical Activity Enjoyment Scale; PBS: Pediatric Balance Scale; PEDI: Pediatric Evaluation of Disability Inventory; PEDI-CAT: Pediatric Evaluation of Disability Inventory-Computer Adaptive Test; PedsQoL: Pediatric Quality of Life Inventory; PMAL: Pediatric Motor Activity Log; PMAL-R: Pediatric Motor Activity Log-Revised; PV: Post-Intervention Evaluation; QOM: Quality of Movement; QUEST: Quality of Upper Extremity Skills Test; RCT: Randomized Controlled Trial; SP: Spain; TCS: Trunk Control Score; TOT: Task-Oriented Training; TR: Turkey; TW: Taiwan; UCP: Unilateral Cerebral Palsy; UE: Upper Extremity Motor Control; USCP: Unilateral Spastic Cerebral Palsy; VR: Virtual Reality; WeeFIM: Functional Independence Measure for Children.

**Table 3:** Effectiveness of occupational therapy-based intervention on gross motor functions in children with cerebral palsy.

Gross Motor Skills									
	N ^a^	N ^b^	N ^c^	N ^d^	ES (95% CI)	*p*	*I*^2^ (%)	Egger’s Test (*p*)	RW (%)
GMFM-66 (pts)	3	3	3	161	0.32 (0.01 to 0.63)	**0.04**	0.00	0.84	4.57–12.9

Bolded *p*-values reflect significant improvements (*p* < 0.05) in the experimental group versus control after occupational therapy. Non-significant values (*p* > 0.05) indicate minimal publication bias. ^a^ Number of studies included; ^b^ experimental groups; ^c^ control groups; ^d^ total participants with cerebral palsy; 95% CI: 95% confidence interval; ES: effect size (Hedges’ g); GMFM-66: gross motor function measure-66; RW: relative weight per study.

**Table 4:** Summary of results of the meta-analysis.

**Functional Ability**									
	**N ^a^**	**N ^b^**	**N ^c^**	**N ^d^**	**ES (95% CI)**	***p***	***I*^2^ (%)**	**Egger’s Test (*p*)**	**RW (%)**
PEDI-Self Care (pts)	4	4	4	127	0.19 (−0.14 to 0.53)	0.26	0.00	0.86	2.00–6.33
PEDI-Mobility (pts)	3	3	3	88	0.46 (0.05 to 0.87)	**0.02**	0.00	0.92	4.99–10.4
**Occupational Performance and Satisfaction**									
	**N ^a^**	**N ^b^**	**N ^c^**	**N ^d^**	**ES (95% CI)**	***p***	***I*^2^ (%)**	**Egger’s Test (*p*)**	**RW (%)**
COPM-P (pts)	5	5	5	163	2.63 (1.14 to 4.11)	**0.001**	92.2	0.00	1.74–4.58
COPM-S (pts)	5	5	5	163	2.17 (0.82 to 3.51)	**0.002**	91.4	0.00	2.11–4.59

Bolded *p*-values indicate significant improvement (*p* < 0.05) in the experimental group post-occupational therapy versus control, while *p* > 0.05 denotes low publication bias. ^a^ Number of studies analyzed; ^b^ Experimental groups; ^c^ Control groups; ^d^ Total participants with cerebral palsy; 95% CI: Confidence interval; ES: Effect size (Hedges’ g); COPM-P: Canadian occupational performance measure-performance; COPM-S: Canadian occupational performance measure-satisfaction; PEDI: Pediatric evaluation of disability inventory; RW: Relative weight per study.

**Table 5:** Evaluation of methodological quality using the GRADEpro tool.

Certainty of Evidence							Nº of Patients		Effect		**Certainty**	**Importance**
Nº of Studies	Study Design	Risk of Bias	Inconsistency	Indirect Evidence	Vagueness	Other Considerations	[Intervention]	[Comparison]	Relative (95% CI)	**Absolute (95% CI)**		
**Gross motor skills**												
3	RCT	Serious	It is not serious	It is not serious	It is not serious	None	80/161 (49.7%)	81/161 (50.3%)	Not estimable		+++ Moderate	IMPORTANT
**Daily living activities—functional ability**												
4	RCT	Serious	It is not serious	It is not serious	It is not serious	None	64/127 (50.4%)	63/127 (49.6%)	Not estimable		+++ Moderate	IMPORTANT
**Daily living activities —occupational performance and satisfaction**												
4	RCT	Serious	It is not serious	It is not serious	It is not serious	None	105/197 (53.3%)	92/197 (46.7%)	Not estimable		+++ Moderate	IMPORTANT

RCT: Randomized clinical trial.

## Supplementary Materials

The following supporting information can be downloaded at https://www.mdpi.com/article/10.3390/jcm14217624/s1, Table S1. PRISMA checklist; Figure S1. Forest plot of the Gross Motor Function Measure (GMFM-66) outcomes. The meta-analysis indicates low heterogeneity among included studies (I^2^ = 0.00%, *p* = 0.84), demonstrating consistent results across trials evaluating motor function after Occupational therapy-based intervention; Figure S2. Forest plot of the Pediatric Evaluation of Disability Inventory—Self-Care (PEDI—Self Care) outcomes. Results show low heterogeneity (I^2^ = 0.00%, *p* = 0.86), indicating stable effects among studies assessing self-care performance improvements; Figure S3. Forest plot of the Pediatric Evaluation of Disability Inventory—Mobility (PEDI—Mobility) outcomes. The analysis shows low heterogeneity (I^2^ = 0.00%, *p* = 0.92), suggesting consistent findings regarding mobility improvements following OT-based rehabilitation; Figure S4. Forest plot of the Canadian Occupational Performance Measure—Performance (COPM—Performance) outcomes. This analysis reveals high heterogeneity (I^2^ = 92.2%, *p* = 0.00), reflecting variability among studies in performance-related outcomes; Figure S5. Forest plot of the Canadian Occupational Performance Measure—Satisfaction (COPM—Satisfaction) outcomes. Results show high heterogeneity (I^2^ = 91.4%, *p* = 0.00), suggesting considerable differences among studies in satisfaction-related measures. Ref. [57] is listed in the supplementary.
