# Supplementary material for: Effectiveness of Occupational Therapy-Based Intervention on Gross Motor Function and Independence in Activities of Daily Living in Children with Cerebral Palsy: A Systematic Review with Meta-Analysis
Source: J Clin Med. 2025 Oct 27;14(21):7624. doi: 10.3390/jcm14217624 (PMC12608141; doi:10.3390/jcm14217624)
Supplement: Supplementary file 1 [file jcm-14-07624-s001.zip › jcm-3909127-supplementary.pdf]

Supplementary Materials

Table S1. PRISMA Checklist.

Section and Topic	Item #	Checklist item	Location where item is reported
**TITLE**			
Title	1	Identify the report as a systematic review.	Lines 2-5
**ABSTRACT**			
Abstract	2	See the PRISMA 2020 for Abstracts checklist.	Lines 37-57
**INTRODUCTION**			
Rationale	3	Describe the rationale for the review in the context of existing knowledge.	Lines 61-113
Objectives	4	Provide an explicit statement of the objective(s) or question(s) the review addresses.	Lines 114-116
**METHODS**			
Eligibility criteria	5	Specify the inclusion and exclusion criteria for the review and how studies were grouped for the syntheses.	Lines 122-129
Information sources	6	Specify all databases, registers, websites, organisations, reference lists and other sources searched or consulted to identify studies. Specify the date when each source was last searched or consulted.	Lines 130-133
Search strategy	7	Present the full search strategies for all databases, registers and websites, including any filters and limits used.	Lines 130-147
Selection process	8	Specify the methods used to decide whether a study met the inclusion criteria of the review, including how many reviewers screened each record and each report retrieved, whether they worked independently, and if applicable, details of automation tools used in the process.	Lines 148-155, Table 1
Data collection process	9	Specify the methods used to collect data from reports, including how many reviewers collected data from each report, whether they worked independently, any processes for obtaining or confirming data from study investigators, and if applicable, details of automation tools used in the process.	Lines 164-174
Data items	10a	List and define all outcomes for which data were sought. Specify whether all results that were compatible with each outcome domain in each study were sought (e.g. for all measures, time points, analyses), and if not, the methods used to decide which results to collect.	Lines 164-174, Table 1
	10b	List and define all other variables for which data were sought (e.g. participant and intervention characteristics, funding sources). Describe any assumptions made about any missing or unclear information.	Lines 164-174
Study risk of bias	11	Specify the methods used to assess risk of bias in the included studies, including details of the tool(s) used, how many reviewers assessed each	Lines 175-

Section and Topic	Item #	Checklist item	Location where item is reported
assessment		study and whether they worked independently, and if applicable, details of automation tools used in the process.	180
Effect measures	12	Specify for each outcome the effect measure(s) (e.g. risk ratio, mean difference) used in the synthesis or presentation of results.	Lines 181-197
Synthesis methods	13a	Describe the processes used to decide which studies were eligible for each synthesis (e.g. tabulating the study intervention characteristics and comparing against the planned groups for each synthesis (item #5)).	Lines 181-197, Figure 1
	13b	Describe any methods required to prepare the data for presentation or synthesis, such as handling of missing summary statistics, or data conversions.	Lines 181-197, Figure 1
	13c	Describe any methods used to tabulate or visually display results of individual studies and syntheses.	Figure 1 to 8
	13d	Describe any methods used to synthesize results and provide a rationale for the choice(s). If meta-analysis was performed, describe the model(s), method(s) to identify the presence and extent of statistical heterogeneity, and software package(s) used.	Lines 181-197, Figure 1
	13e	Describe any methods used to explore possible causes of heterogeneity among study results (e.g. subgroup analysis, meta-regression).	Lines 181-197
	13f	Describe any sensitivity analyses conducted to assess robustness of the synthesized results.	Lines 181-197
Reporting bias assessment	14	Describe any methods used to assess risk of bias due to missing results in a synthesis (arising from reporting biases).	Lines 198-207
Certainty assessment	15	Describe any methods used to assess certainty (or confidence) in the body of evidence for an outcome.	Lines 198-207
**RESULTS**			
Study selection	16a	Describe the results of the search and selection process, from the number of records identified in the search to the number of studies included in the review, ideally using a flow diagram.	Lines 209-221, Figure 1
	16b	Cite studies that might appear to meet the inclusion criteria, but which were excluded, and explain why they were excluded.	Lines 209-221, Figure 1
Study characteristics	17	Cite each included study and present its characteristics.	Lines 235-247, Table 2
Risk of bias in	18	Present assessments of risk of bias for each included study.	Lines 226-

Section and Topic	Item #	Checklist item	Location where item is reported
studies			234, Figure 2, Figure 3
Results of individual studies	19	For all outcomes, present, for each study: (a) summary statistics for each group (where appropriate) and (b) an effect estimate and its precision (e.g. confidence/credible interval), ideally using structured tables or plots.	Lines 261-323, Table 2 to 4
Results of syntheses	20a	For each synthesis, briefly summarise the characteristics and risk of bias among contributing studies.	Figures 4 to 8; Figure S1 to S5
	20b	Present results of all statistical syntheses conducted. If meta-analysis was done, present for each the summary estimate and its precision (e.g. confidence/credible interval) and measures of statistical heterogeneity. If comparing groups, describe the direction of the effect.	Figures 4 to 8, Figure S1 to S5
	20c	Present results of all investigations of possible causes of heterogeneity among study results.	Figures 4 to 8, Figure S1 to S5
	20d	Present results of all sensitivity analyses conducted to assess the robustness of the synthesized results.	Figures 4 to 8, Figure S1 to S5
Reporting biases	21	Present assessments of risk of bias due to missing results (arising from reporting biases) for each synthesis assessed.	Not applicable
Certainty of evidence	22	Present assessments of certainty (or confidence) in the body of evidence for each outcome assessed.	Lines 324-328
**DISCUSSION**			
Discussion	23a	Provide a general interpretation of the results in the context of other evidence.	Lines 334-364
	23b	Discuss any limitations of the evidence included in the review.	Lines 365-370
	23c	Discuss any limitations of the review processes used.	Lines 365-370
	23d	Discuss implications of the results for practice, policy, and future research.	Lines 377-389
**OTHER INFORMATION**			
Registration and	24a	Provide registration information for the review, including register name and registration number, or state that the review was not registered.	Lines 44, 121 and

Section and Topic	Item #	Checklist item	Location where item is reported
protocol			183
	24b	Indicate where the review protocol can be accessed, or state that a protocol was not prepared.	Lines 44, 121 and 183
	24c	Describe and explain any amendments to information provided at registration or in the protocol.	Not apply
Support	25	Describe sources of financial or non-financial support for the review, and the role of the funders or sponsors in the review.	Not apply
Competing interests	26	Declare any competing interests of review authors.	Lines 425
Availability of data, code and other materials	27	Report which of the following are publicly available and where they can be found: template data collection forms; data extracted from included studies; data used for all analyses; analytic code; any other materials used in the review.	Not apply

From: Page MJ, McKenzie JE, Bossuyt PM, Boutron I, Hoffmann TC, Mulrow CD, et al. The PRISMA 2020 statement: an updated guideline for reporting systematic reviews. BMJ 2021;372:n71. doi: 10.1136/bmj.n71. This work is licensed under CC BY 4.0. To view a copy of this license, visit https://creativecommons.org/licenses/by/4.0/

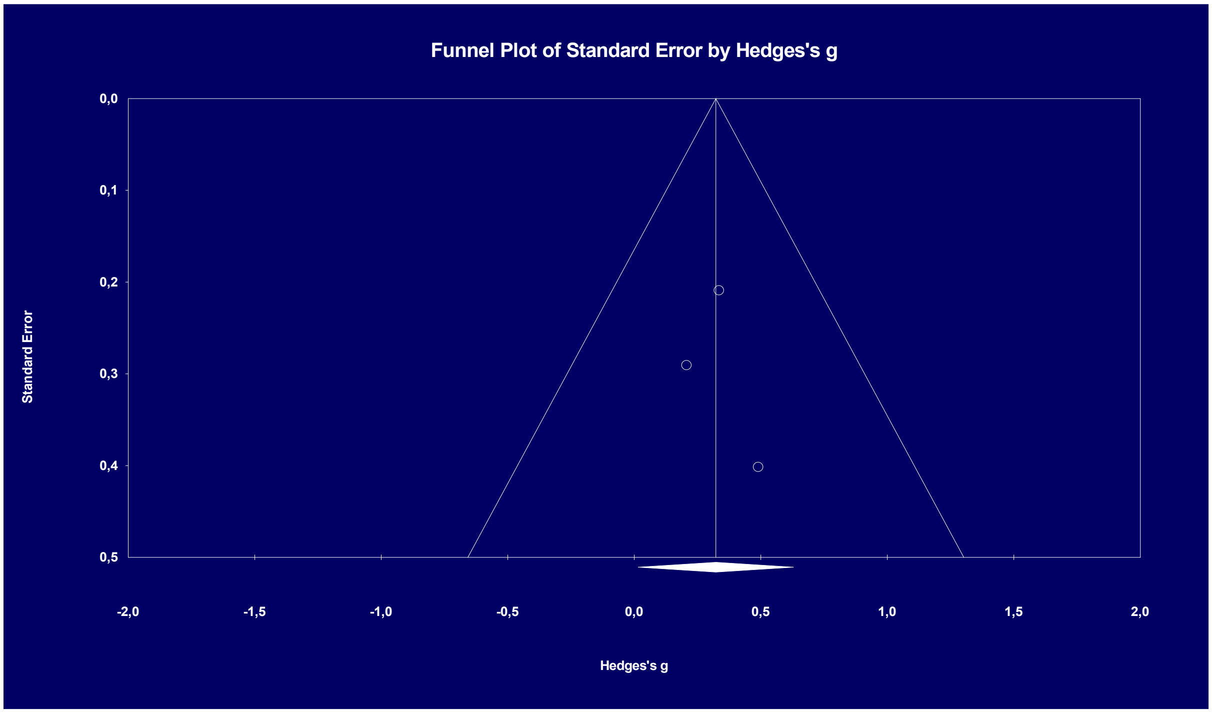

Figure S1. Forest plot of the Gross Motor Function Measure (GMFM-66) outcomes. The meta-analysis indicates low heterogeneity among included studies ($I^2 = 0.00\%$, $p = 0.84$), demonstrating consistent results across trials evaluating motor function after Occupational therapy-based intervention.

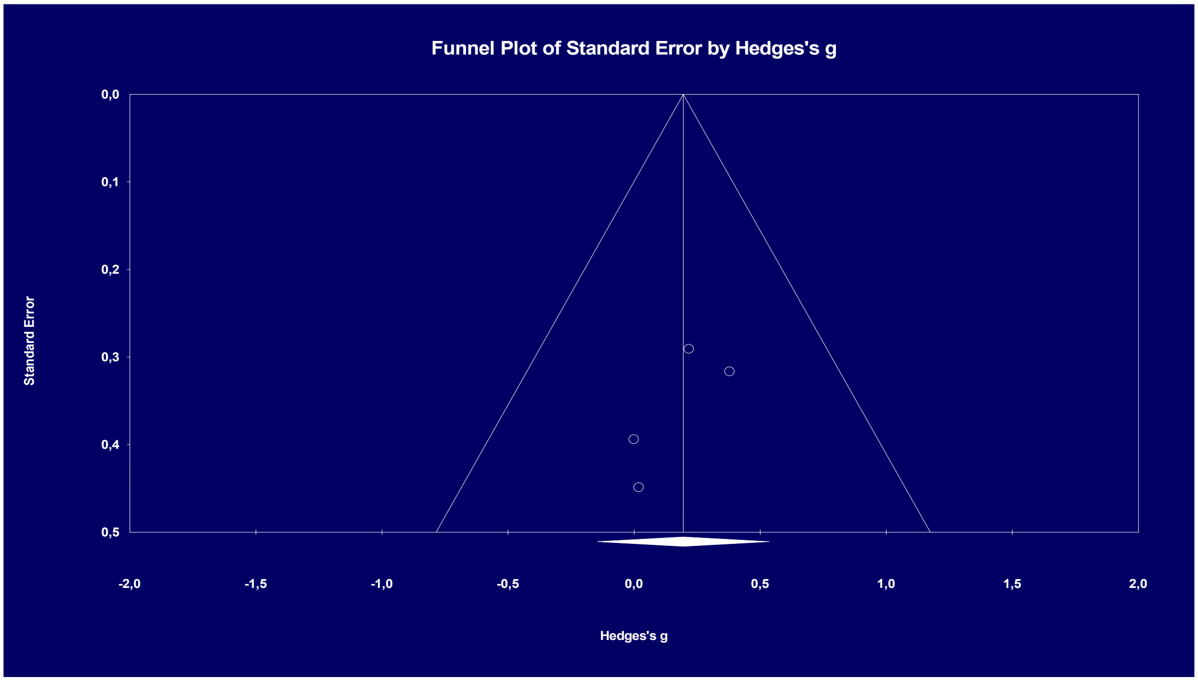

Figure S2. Forest plot of the Pediatric Evaluation of Disability Inventory – Self-Care (PEDI–Self Care) outcomes. Results show low heterogeneity ($I^2 = 0.00\%$, $p = 0.86$), indicating stable effects among studies assessing self-care performance improvements.

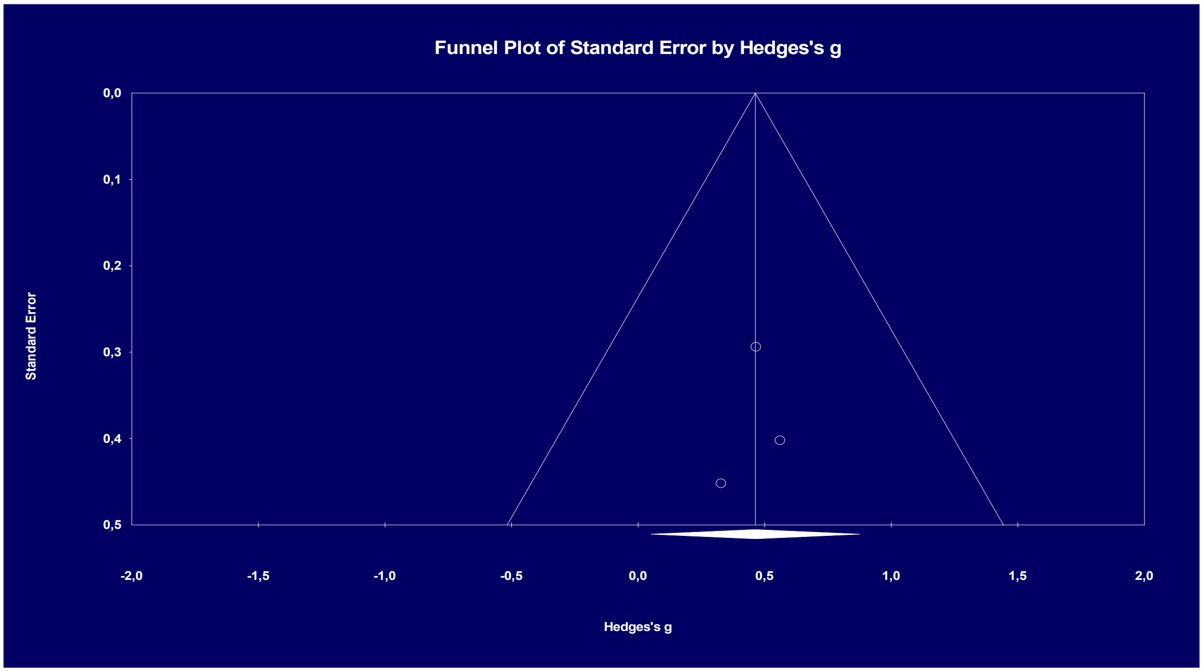

Figure S3. Forest plot of the Pediatric Evaluation of Disability Inventory – Mobility (PEDI–Mobility) outcomes. The analysis shows low heterogeneity (I^2 = 0.00%, p = 0.92), suggesting consistent findings regarding mobility improvements following OT-based rehabilitation.

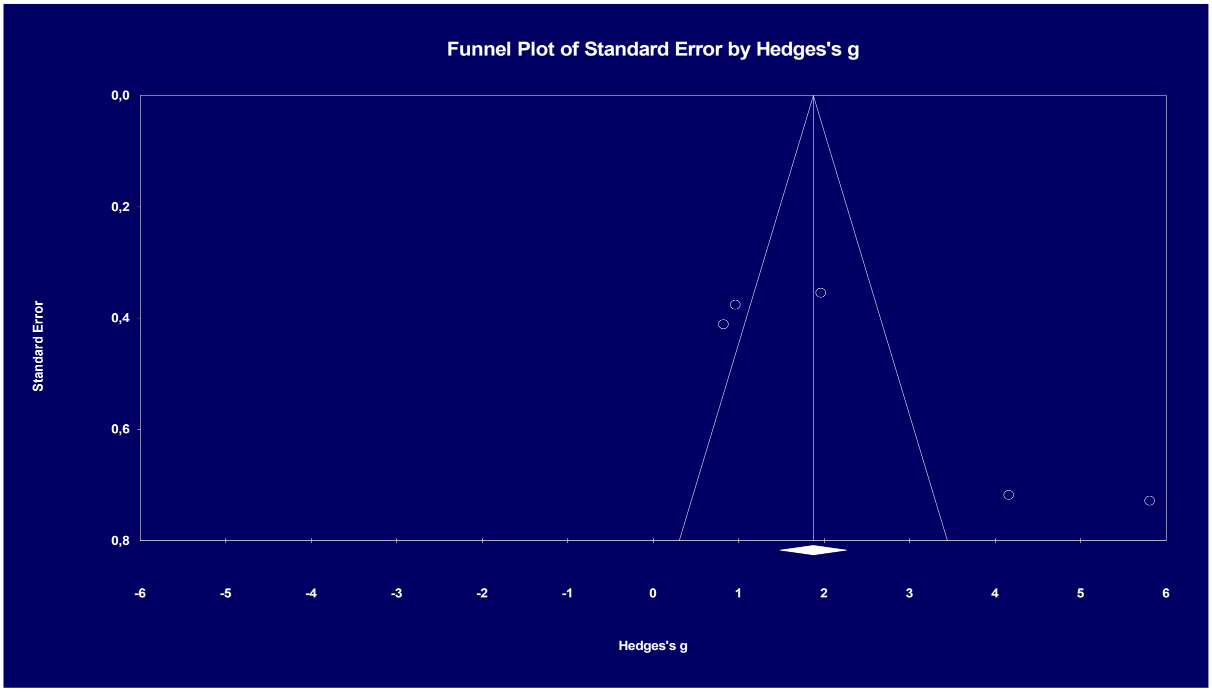

Figure S4. Forest plot of the Canadian Occupational Performance Measure – Performance (COMP–Performance) outcomes. This analysis reveals high heterogeneity (I^2 = 92.2%, p = 0.00), reflecting variability among studies in performance-related outcomes.

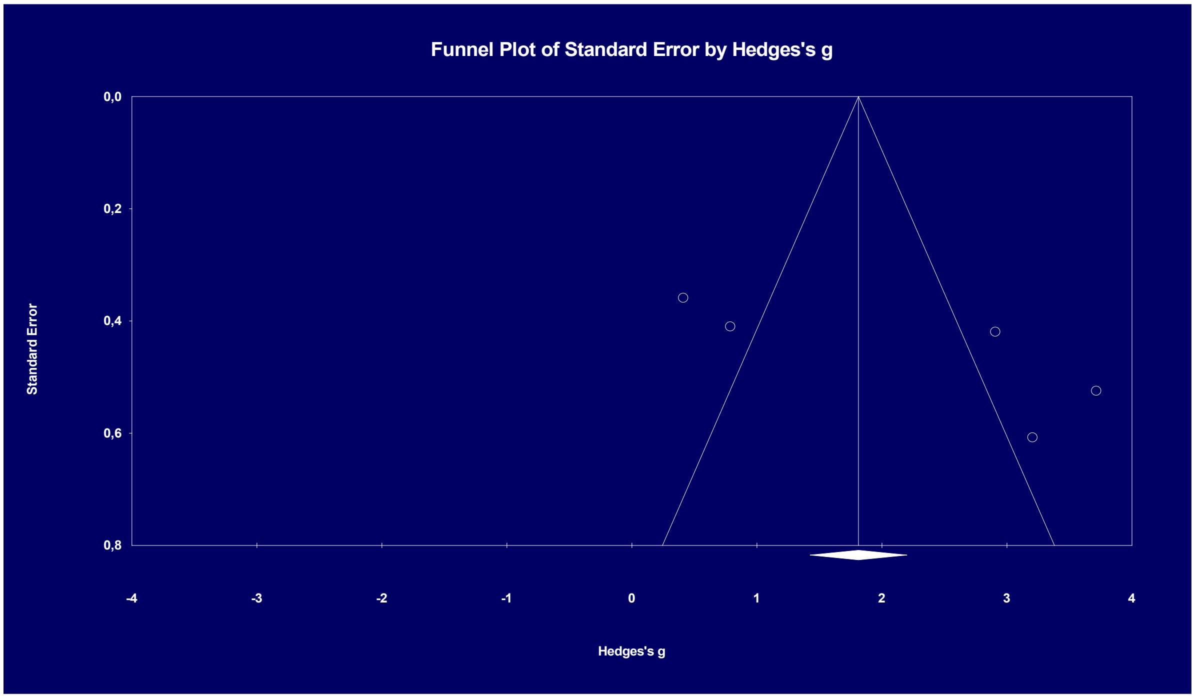

Figure S5. Forest plot of the Canadian Occupational Performance Measure – Satisfaction (COMP–Satisfaction) outcomes. Results show high heterogeneity (I^2 = 91.4%, p = 0.00), suggesting considerable differences among studies in satisfaction-related measures**.**
